# Pegylated liposomal doxorubicin in patients with epithelial ovarian cancer

**DOI:** 10.1186/s13048-020-00736-2

**Published:** 2021-01-11

**Authors:** Zhen Yuan, Ying Zhang, Dongyan Cao, Keng Shen, Qingshui Li, Guonan Zhang, Xiaohua Wu, Manhua Cui, Ying Yue, Wenjun Cheng, Li Wang, Pengpeng Qu, Guangshi Tao, Jianqing Hou, Lixin Sun, Yuanguang Meng, Guiling Li, Changzhong Li, Huirong Shi, Yaqing Chen

**Affiliations:** 1 Department of Obstetrics and Gynecology, Peking Union Medical College Hospital, Peking Union Medical College, Chinese Academy of Medical Sciences, Beijing, Zip code: 100730 China; 2 Department of Gynecologic Oncology, Shandong Cancer Hospital & Institute, Shandong, China; 3 Department of Gynecologic Oncology, Sichuan Cancer Hospital & Institute, Sichuan, China; 4 Department of Gynecologic Oncology, Fudan University Shanghai Cancer Center, Shanghai, China; 5 Department of Obstetrics and Gynecology, The Second Hospital of Jilin University, Jilin, China; 6 Department of Obstetrics and Gynecology, The First Bethune Hospital of Jilin University, Jilin, China; 7 Department of Obstetrics and Gynecology, Jiangsu Province Hospital, Jiangsu, China; 8 Department of Gynecologic Oncology, He Nan Cancer Hospital, Henan, China; 9 Department of Obstetrics and Gynecology, Tianjin Central Hospital of Gynecology Obstetrics, Tianjin, China; 10 Department of Obstetrics and Gynecology, The Second Xiangya Hospital of Central South University, Hunan, China; 11 Department of Obstetrics and Gynecology, Yantai Yuhuangding Hospital, Shandong, China; 12 Department of Gynecologic Oncology, Shanxi Cancer Hospital, Shanxi, China; 13 Department of Obstetrics and Gynecology, Chinese PLA General Hospital, Beijing, China; 14 Department of Obstetrics and Gynecology, Wuhan Union Hospital of China, Hubei, China; 15 Department of Obstetrics and Gynecology, Shandong Provincial Hospital, Shandong, China; 16 Department of Obstetrics and Gynecology, The First Affiliated Hospital of Zhengzhou University, Henan, China; 17 Department of Gynecologic Oncology, Zhejiang Cancer Hospital, Zhejiang, China

**Keywords:** CA125, Pegylated liposomal doxorubicin, Platinum-refractory relapse, Platinum-resistant relapse, Partially platinum-sensitive relapse

## Abstract

**Objective:**

To evaluate the efficacy and safety of PLD in treating of in patients who experience epithelial ovarian, fallopian tubal, and peritoneal cancer progression within 12 months after the first-line platinum-based therapy.

**Methods:**

This was an open-label, single-arm and multicenter clinical trial. The ORR was the interim primary objective, and the DCR, AEs and QOL were the secondary objectives. The impact of factors on efficacy outcomes, the change trend of CA125 and the artificial platinum-free interval were exploratory endpoints.

**Results:**

Totally, 115 patients were enrolled in this study and included in the ITT population. Moreover, 101 patients were included in the safety population. The median follow-up time was 4 months (IQR 2–6). In the ITT population, the confirmed ORR was 37.4% (95% CI, 28.4–46.4%), and the DCR was 65.2% (95% CI, 56.4–74.1%). The previous response status to platinum-based chemotherapy and baseline CA125 levels were significantly correlated with the ORR. The ORR was significantly higher in patients with a CA125 decrease after the first cycle than in the patients with a CA125 increase. The most common grade 3 or higher AE was hand-foot syndrome (3 [3.0%] of 101 patients). No statistically significant differences existed between the baseline and the postbaseline questionnaires.

**Conclusions:**

For patients who experience platinum-resistant and platinum-refractory relapse, the use of PLD may be acceptable because of the associated satisfactory efficacy, low frequency of AEs and high patient QOL. Moreover, a low CA125 level at baseline and a reduction in CA125 after the first cycle are predictive factors for satisfactory efficacy.

**Supplementary Information:**

The online version contains supplementary material available at 10.1186/s13048-020-00736-2.

## Introduction

In 2018, it was estimated that 22,240 new diagnoses of ovarian cancer occurred in the United States [1]. More than 70% of patients present with advanced disease [2]. Approximately 80% of patients with advanced ovarian cancer will experience tumor progression or relapse [3]. Half of all first relapses occur within 12 months after ending first-line therapy, and one-quarter of all relapses occur within 6 months [4], which is defined as platinum-refractory or platinum-resistant relapse. The current management and treatment options for platinum-resistant and platinum-refractory recurrent ovarian cancer are limited [5]. A retrospective study described the real-world treatment patterns in these patients from January 2010 to June 2014 in the United States, the United Kingdom, and Canada and found that the most common initial therapy was pegylated liposomal doxorubicin (PLD) monotherapy [6].

PLD is a complex formulation of doxorubicin based on pharmaceutical nanotechnology with unique pharmacokinetic and pharmacodynamic properties. Since PLD has a long circulation time and stable retention of the payload and accumulates in tumors with high vascular permeability, this drug has important advantages over conventional chemotherapies [7].

For patients who experience partially platinum-sensitive relapse, which is defined as progression within 6 to 12 months after the last platinum-based chemotherapy treatment, the treatment has not yet been standardized [3, 8–11], and there are certain factors that prevent some patients from re-using platinum-based chemotherapy shortly after the frontline platinum-based chemotherapy [12].

Therefore, this clinical trial was aimed to evaluate the efficacy and safety of PLD in treating patients who experience platinum-refractory, platinum-resistant and partially platinum-sensitive relapse in China.

## Methods

### Study design

This was an open-label, single-arm and multicenter prospective clinical trial conducted in China. This trial was designed to evaluate the efficacy and safety of Chinese-made PLD in patients who experienced epithelial ovarian, tubal, and peritoneal cancer progression or relapse within 12 months after finishing the first-line platinum-based chemotherapy. This study was registered in the Chinese Clinical Trial Registry under the number is ChiCTR1900022962. All procedures performed in studies involving human participants were in accordance with the ethical standards. Informed consent was obtained from all individual participants included in the study.

### Participants

Women aged 18–80 years from seventeen medical centers were included who experienced progression during primary platinum-based chemotherapy or first relapsed within 12 months after the last chemotherapy treatment were included. A histologically confirmed diagnosis of epithelial ovarian cancer, fallopian tubal or peritoneal epithelial cancer was required. As required, primary treatment included only one line of platinum-based chemotherapy (paclitaxel and carboplatin or cis-platinum) and no second-line chemotherapy treatments. Patients were required to have measurable disease by the Response Evaluation Criteria in Solid Tumors (RECIST version 1.1) or according to the Gynecologic Cancer Intergroup (GCIG) criteria as assessed by serum cancer antigen (CA) 125 levels. The other key eligibility criteria were as follows: an Eastern Cooperative Oncology Group (ECOG) performance status of 0–2, a life expectancy of at least 3 months, adequate heart function (left ventricular ejection fraction ≥50% on echocardiogram), adequate bone marrow function (absolute neutrophil count ≥1500 cells per μL, platelet count ≥80,000 cells per μL, and hemoglobin concentration ≥ 80 g/dL), adequate liver function (alanine aminotransferase or aspartate aminotransferase ≤2.5-fold the upper limit of normal and total bilirubin ≤2.5-fold the upper limit of normal), and adequate renal function (creatinine ≤1.5-fold the upper limit of normal). The key exclusion criteria were as follows: primary treatment with only chemotherapy agents without cytoreductive surgery; previous pelvic or abdominal radiotherapy; brain metastasis; acute infection; history of a secondary malignancy in the past 5 years; a total cumulative dose of doxorubicin ≥300 mg/m^2^; a cumulative dose of epirubicin ≥550 mg/m^2^ and cardiac lesions caused by anthracyclines.

### Procedures

PLD (40 mg/m^2^) was administered intravenously and repeated every 4 weeks. Treatment was administered for 6 cycles, and fewer cycles were administered if the patients experienced disease progression or unacceptable toxicity, if the local investigator decided to reduce the number of cycles, or if the patient withdrew consent. After 6 cycles of chemotherapy, the prescription of additional cycles was allowed based on the local investigator’s decision. Disease was assessed by computed tomography (CT) scans or CA125 levels according to the RECIST version 1.1 or the GCIG criteria, respectively, and these assessments were performed at baseline, 3–4 weeks after every 2 cycles, and 4 weeks after the last treatment. Moreover, CA125 levels were measured at baseline and within 3 days before each cycle. The safety assessment, which included a physical examination, blood tests (hematology and biochemistry), and history of adverse events (AEs), was performed at baseline, before each cycle, and 4 weeks after the end of treatment. Hematology was assessed weekly. Electrocardiography and echocardiography were planned at baseline and after every 2 cycles. AEs were recorded and graded according to the Common Terminology Criteria for Adverse Events (CTCAE) version 4.03. The European Organization for Research and Treatment of Cancer Core Quality of Life Questionnaire (EORTC QLQ-C30) was used to evaluate quality of life (QOL) at baseline and within 3 days before the third and fifth cycles.

### Outcomes

The primary mid-term research objective was the objective response rate (ORR), which was defined as the rate of complete remission (CR) and partial remission (PR). The secondary mid-term objectives were the disease control rate (DCR), which was defined as the rate of CR or PR and stable disease (SD), safety and QOL. The disease response was assessed by investigators according to the RECIST1.1 and the GCIG criteria [13, 14]. The impact of factors on the efficacy outcomes, the change trend of CA125 and the time interval between last platinum-based chemotherapy or the enrollment and the time at which a patient was switched to another therapy (artificial platinum-free interval) were the exploratory endpoints.

For the patients with partially platinum-sensitive relapse, there was no power analysis. Patients with platinum-refractory or resistant relapse were assessed with a Simon’s two-stage design with a two-sided an error of 5% and a power of 80% [15]. Previous studies indicated that the ORR of PLD monotherapy in patients with platinum- refractory or resistant relapse was 15–40.4% [6, 16], and we initially expected an ORR of 30% for PLD in these patients. Therefore, we set P0 to 15%, and P1 to 30% in this study. Under these assumptions, twenty-three patients needed to be treated in the first stage, and at least 3 responses were required to continue to the second stage. Forty-eight patients would be enrolled in the second stage, for a total sample size of 71, and if 11 or more responses were observed, the treatment regimen would be considered a success. Moreover, considering a 10% rate of loss to follow up, the total sample size was 78.

The analyses were performed on three populations: the intention-to- treat (ITT) population, the per-protocol (PP) population, and the safety population. The ITT population included all enrolled patients. The PP population was a subgroup of patients who met all of the trial criteria and were compliant with the protocol, did not have any major protocol violations, and had at least one post-baseline efficacy assessment. The safety population included the enrolled patients who received at least one cycle of PLD and had available surveillance data. We analyzed efficacy in the ITT and PP populations, and safety in the safety population.

### Statistical analysis

Categorical variables are summarized in frequency tables, whereas continuous variables are presented as the mean ± standard deviation or median (interquartile range (IQR), range), as appropriate for data distribution. Frequency distributions were compared using Pearson’s chi-square test or the likelihood ratio, as appropriate. Between two groups, mean values were compared using t-tests and median values were compared using a non-parametric test. QOL subscales were summarized using the mean and 95% confidence interval (CI), and one-way ANOVA was used to compare the mean values between multiple groups. Binary logistic regression was used to explore the impact of factors on efficacy. Variates with *P* < 0.1 in the univariate analysis were entered into the multivariate analysis. The time interval between enrollment and the time at which a patient was switched to another therapy was analyzed using the Kaplan–Meier method. We analyzed data that were collected by the cutoff date of June 2, 2019. The data were analyzed using SPSS (version 23, IBM, Armonk, NY) or Prism 7 (GraphPad 66 Software, San Diego, CA). A *P* value < 0.05 was considered statistically significant (two-tailed hypothesis).

## Results

**Fig. 1:**
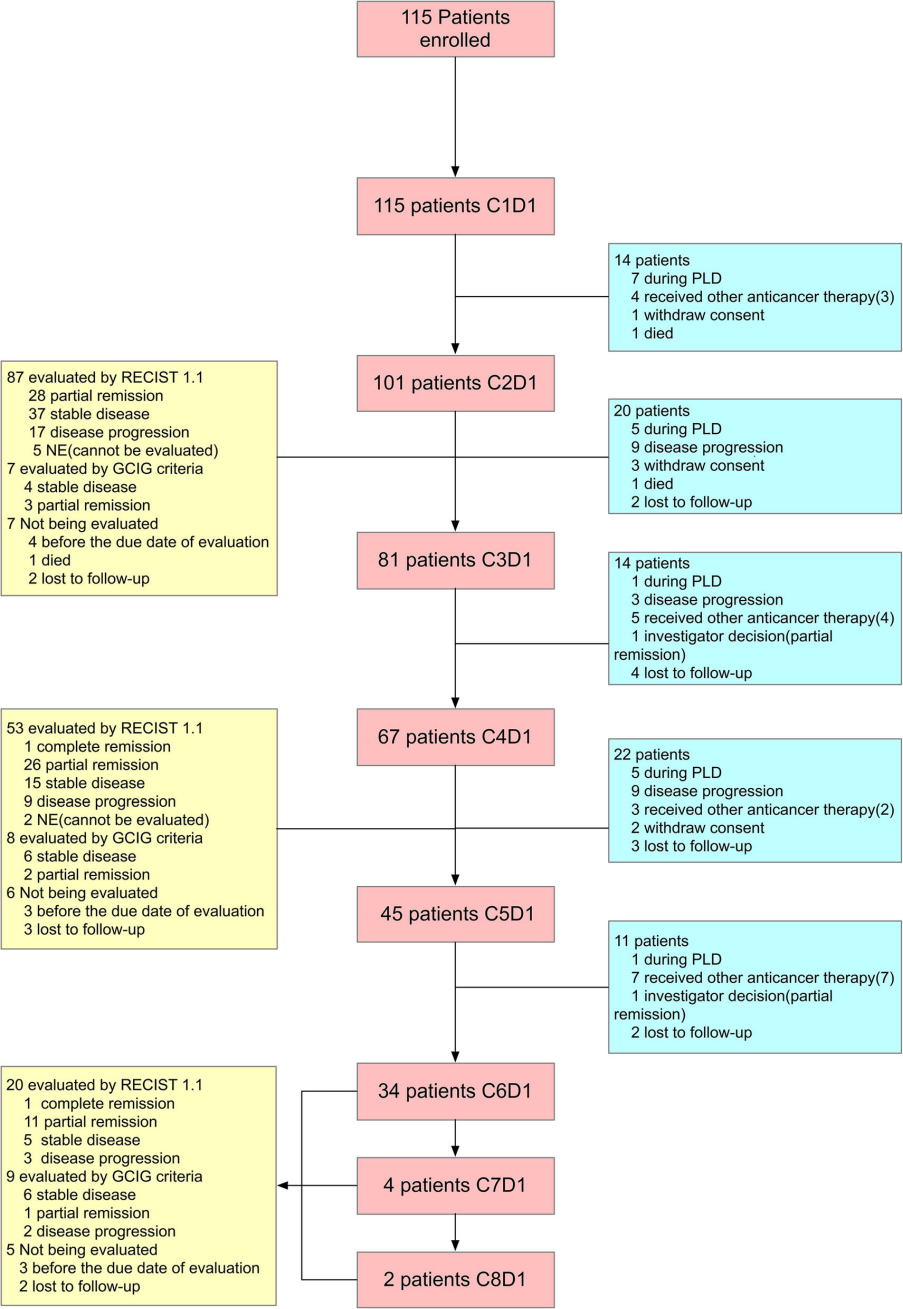
The clinical trial profile. C, cycle; D, day; PLD, pegylated liposomal doxorubicin; RECIST 1.1, the Response Evaluation Criteria in Solid Tumors version 1.1; GCIG, Gynecologic Cancer InterGroup

Between June 2017 and June 2019, 115 patients were enrolled in this study (Fig. 1; Table 1.) and included in the ITT analysis. Ninety-two patients with a confirmed post-baseline efficacy assessment were included in the PP population. A total of 101 patients were included in the safety analysis.

**Table 1:** Baseline patient characteristics.

Overall	All patients enrolled(***N*** = 115)
Age, years	53.53(±8.69)
**Data for front-line treatment in this study**	
**The tumor origin**	
Ovary	107 (93.0%)
Fallopian tube	6 (5.2%)
Peritoneum	1 (0.9%)
unknown	1 (0.9%)
**Pathologic histology type**	
Serous Tumors	108 (93.9%)
High-grade	91 (84.3%)
Low-grade	8 (7.4%)
Unknown	9 (8.3%)
Mucinous Tumors	1 (0.9%)
Endometrioid Tumors	1 (0.9%)
Clear Cell Tumors	4 (3.5%)
Mixed epithelial tumors	1 (0.9%)
**FIGO stage**	
I	1 (0.9%)
II	4 (3.5%)
III	97 (84.3%)
IIIA	10 (10.3%)
IIIB	10 (10.3%)
IIIC	74 (76.3%)
Unknown	3 (3.1%)
IV	13 (11.3%)
IVA	3 (23.1%)
IVB	6 (46.2)
Unknown	4 (30.7%)
**Primary cytoreductive surgery**	59 (51.3%)
**Interval cytoreductive surgery**	56 (48.7%)
**Residual disease**	
Optimal cytoreductive surgery	
No gross residual disease (R0)	50 (43.5%)
<1 cm but visible residual disease	46 (40.0%)
Suboptimal cytoreductive	16 (13.9%)
Unknown	3 (2.6%)
**Previous chemotherapy cycles**	
< 6	16 (13.9%)
6–9	92 (80.0%)
> 9	2 (1.7%)
Unknown	5 (4.3%)
**The previous response status to platinum-based chemotherapy**	
Platinum-refractory	36 (31.3%)
Platinum-resistant relapse	31 (27.0%)
Partially platinum- sensitive relapse	48 (41.7%)
**Data before this study**	
**CA125, U/ml**	183.90 (72.27–430.65; 6.00–7135.00)
**ECOG**	
0	63 (54.8%)
1	46 (40.0%)
2	6 (5.2%)

Data are shown as mean (±standard deviation) or median (IQR; range) or n (%). FIGO, International Federation of Gynecology and Obstetrics; ECOG, Eastern Cooperative Oncology Group

The median follow-up time (data cutoff was on June 2, 2019) was 4 months (IQR 2–6 months, range 1–22 months). At the data cutoff point, 22 patients were still receiving treatment (Fig. 1). Fifteen patients (13.0%), including 2 patients (1.7%) who died before the efficacy evaluation during treatment, were lost to follow-up, and 6 patients (5.2%) withdrew consent. Twenty-one patients (18.3%) discontinued PLD because of progressive disease (PD), and 19 patients (16.5%) received other anticancer therapies based on the decision of the investigator, 16 patients of whom (84.2%) received platinum-based chemotherapy.

### Efficacy evaluation

In the ITT analysis, the confirmed ORR was 37.4% (95% CI, 28.4–46.4%): as best responses, 2 patients (1.73%) had confirmed CR, and 41 patients (35.65%) had PR, with a DCR of 65.2% (95% CI, 56.4–74.1%). Of the 43 patients with a confirmed objective response, 31 (72.1%) achieved a confirmed objective response after 2 cycles of PLD, 9 (20.9%) after 4 cycles and 3 patients (7.0%) after 6 cycles. Fourteen patients who received fewer than 2 cycles of PLD and 7 patients who did not undergo efficacy assessments after 2 cycles of PLD were excluded from the PP population. Moreover, 2 patients had post-baseline efficacy assessments that could not be confirmed and thus were excluded. In the PP analysis, the ORR was 46.7% (95% CI, 36.3–57.1%), and the DCR was 81.5% (95% CI, 73.4–89.6%) (Table 2).

**Table 2:** The efficacy analysis

Best Overall Response	Intention-to-treat population(***n*** = 115)	Per-protocol population (***N*** = 92^**a**^)
Complete remission, No. (%)	2 (1.73%)	2 (21.7%)
Partial remission, No. (%)	41 (35.65%)	41 (44.5%)
Stable disease, No. (%)	32 (27.82%)	32 (34.7%)
Disease progression No. (%)	17 (14.7%)	17 (18.4%)
ORR^b^, (95%CI)	37.4% (28.4–46.4%)	46.7% (36.3–57.1%)
DCR^c^, (95%CI)	65.2% (56.4–74.1%)	81.5% (73.4–89.6%)

*ORR* Objective response rate, *DCR* Disease control rate; ^a^ Fourteen patients receiving less than 2 cycles of PLD and 7 patients without efficacy assessments after 2 cycles of PLD were excluded. Moreover,2 patients had a postbaseline efficacy assessment that could not be confirmed and thus were excluded; ^b^ Including patients with complete and partial responses; ^c^ Including patients with complete and partial responses and stable disease

### Exploratory analyses

The outcomes of the exploratory analyses examining the predictive impact of the following factors on efficacy in the PP population using binary logistic regression are presented in Table 3.: age, ECOG performance status, histology, International Federation of Gynecology and Obstetrics (FIGO) stage, neoadjuvant therapy, residual tumor during the initial surgery, response status to platinum-based chemotherapy and CA125 level. The response status to platinum-based chemotherapy and baseline CA125 levels were significantly correlated with the ORR (Table 3.).

**Table 3:** The predictive impact of factors on efficacy

The impact factors of Objective response	Univariate analysis		Multivariate analysis	
	***P***-value	OR (95%CI)	***P***-value	OR (95%CI)
Age	0.511			
ECOG	0.812			
Histology	0.952			
FIGO stage	0.765			
Neoadjuvant therapy	0.706			
Residual tumor	0.296			
Response status to platinum	0.036		0.017	
Resistant VS. Refractory	0.033	3.694 (1.114–12.250)	0.014	5.241 (1.404–19.566)
Partially sensitive VS. Refractory	0.013	4.046 (1.339–12.227)	0.007	5.308 (1.591–17.713)
Baseline CA125	0.077		0.030	
200–500 VS. ≤200	0.146	0.491 (0.188–1.281)	0.033	0.317 (0.110–0.911)
≥500 VS. ≤200	0.039	0.260 (0.072–0.934)	0.029	0.219 (0.056–0.875)

*ECOG* Eastern Cooperative Oncology Group, *FIGO* International federation of Gynecology and Obstetrics

**Fig. 2:**
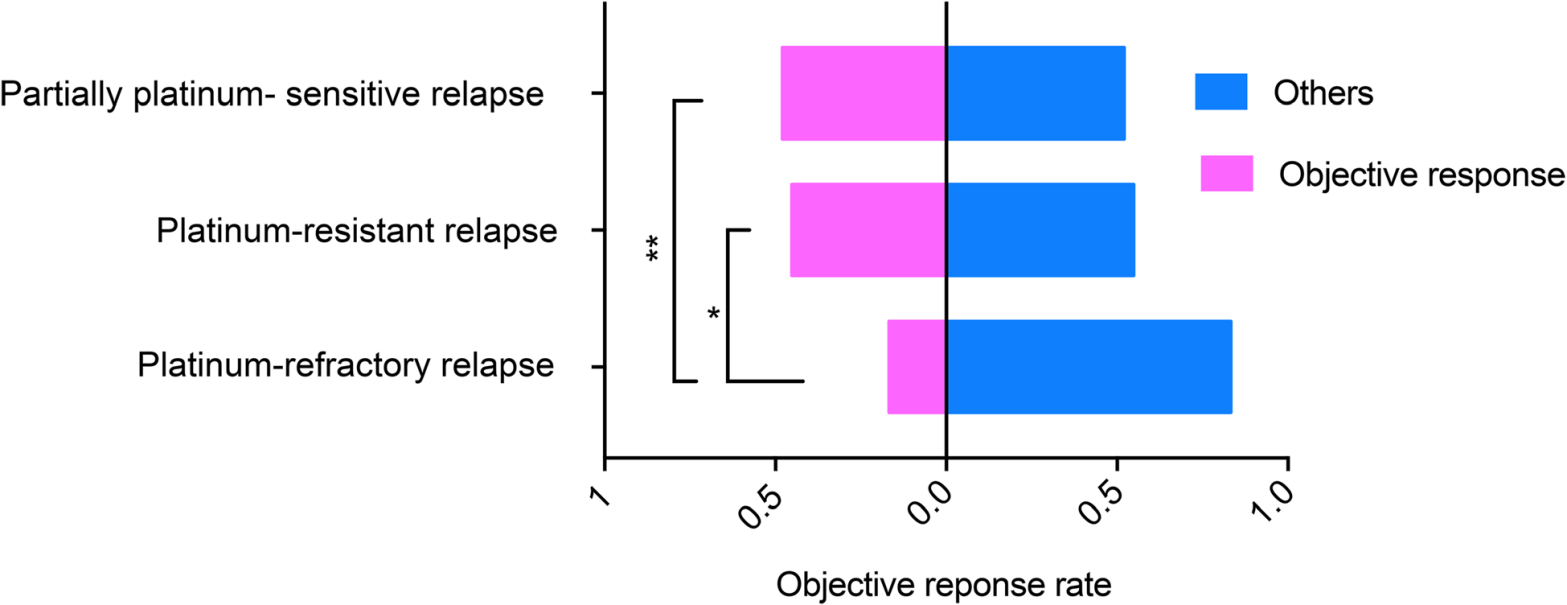
The efficacy analysis based on the response status to platinum-based chemotherapy

Table 4. and Fig. 2 show detailed efficacy results based on the response status to platinum-based chemotherapy. The ORRs in patients with platinum-refractory and -resistant relapse were 16.7 and 45.2% respectively. In addition, of 67 patients with platinum refractory or resistant relapse, 20 patients achieved an objective response, and the total ORR was 29.9% (95% CI, 18.6–41.1%). Moreover, considering the biological differences between different pathologic histology types, the efficacy analysis was performed only on patients with only high-grade serous cancer, as shown in Supplementary Table 1.

**Table 4:** The efficacy analysis based on the response status to platinum-based chemotherapy

	Intention-to-treat population (***N*** = 115)			Per-protocol population (***N*** = 92^**a)**^		
	Platinum-refractory (*N* = 36)	Platinum-resistant (*N* = 31)	Partial platinum-sensitive (*N* = 48)	Platinum-refractory (*N* = 25)	platinum-resistant (*N* = 26)	Partially platinum-sensitive (*N* = 41)
Complete remission, No. (%)	0, (0.0%)	0, (0.0%)	2, (4.2%)	0, (0.0%)	0, (0.0%)	2, (4.9%)
Partial remission, No. (%)	6, (16.7%)	14, (45.2%)	21, (43.8%)	6, (24.0%)	14, (53.8%)	21, (51.2%)
Stable disease, No. (%)	14, (38.9%)	6, (19.4%)	12, (25.0%)	14, (56.0%)	6, (23.1%)	12, (29.3%)
Disease progression, No. (%)	5, (13.9%)	6, (19.4%)	6, (12.5%)	5, (20.0%)	6, (23.1%)	6, (14.6%)
ORR^b^, (95%CI)	16.7% (3.9–29.5%)	45.2% (26.6–63.7%)	47.9% (33.3–62.6%)	24.0% (6.0–42.0%)	53.8% (33.3–74.4%)	56.1% (40.2–72.0%)
DCR^c^, (95%CI)	55.6% (38.5–72.6%)	64.5% (46.7–82.4%)	72.9% (59.9–86.0%)	80.0% (63.1–96.9%)	76.9% (59.6–94.3%)	85.4%(74.1–96.7%)

*ORR* Objective response rate, *DCR* Disease control rate; ^a^ Fourteen patients receiving less than 2 cycles of PLD and 7 patients without efficacy assessments after 2 cycles of PLD were excluded. Moreover,2 patients had a postbaseline efficacy assessment that could not be confirmed and thus were excluded; ^b^ Including patients with complete and partial responses; ^c^ Including patients with complete and partial responses and stable disease

**Fig. 3:**
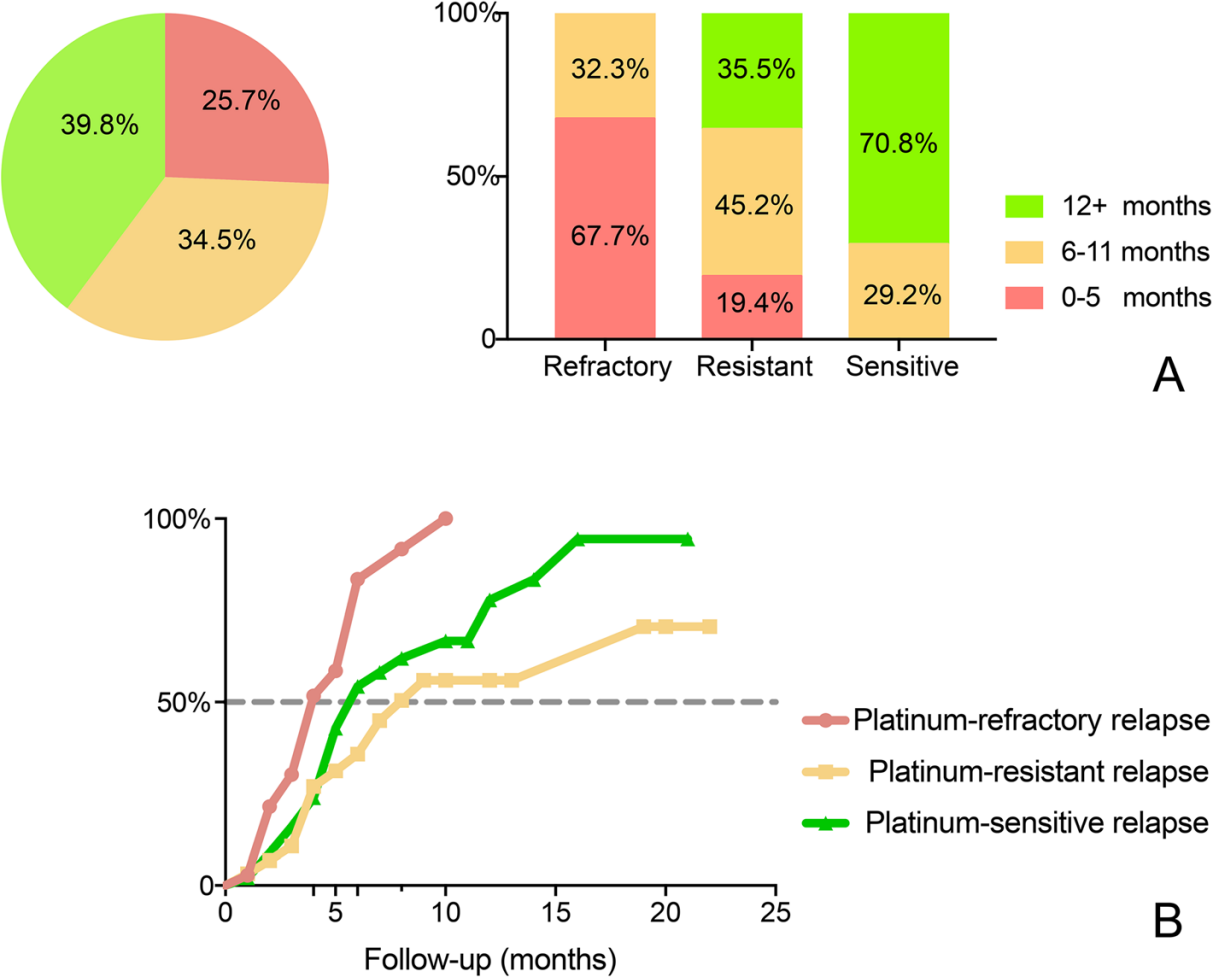
The prolonged platinum-free interval. **a** The time interval between the date of the last first-line platinum-based chemotherapy treatment and the date of changing to other therapies after PLD. **b** The proportion of patients changing to other therapies over time (from the date of enrollment)

This is the preliminary analysis of the response data and the survival data have not been completely analyzed. In addition, many patients in this study switched to other anticancer therapies based on the decision of the investigator, not because of PD. Therefore, we analyzed the time interval between the date of the last first-line platinum-based chemotherapy treatment and the date at which the patient was switched to another therapy after PLD (Fig. 3a) and the time interval between the date of enrollment and the date at which the patient was switched to another therapy (Fig. 3b). The time interval from the date of the last platinum-based chemotherapy treatment to the date of changing to another therapy after PLD was divided into 3 periods: 0–5 months, 6–11 months, and 12 months or more months. In total, PLD treatment prolonged the platinum-free time intervals to at least 12 months in 39.9% of patients and to 6–11 months in 34.5% of patients. Only 25.7% of patients failed to prolong the platinum-free interval to at least 6 months. For the patients with platinum-refractory and-resistant relapse, PLD treatment prolonged the platinum-free interval to at least 6 months in 32.3 and 80.7%, respectively. Figure 3b shows the proportion of patients who were switched to another therapy over time. The median time intervals for patients with platinum-refractory relapse, platinum-resistant relapse and platinum-sensitive relapse were 4 (3–6), 8 (4-~) and 6 (5–12) months, respectively (refractory vs. resistant, *P* = 0.018; refractory vs. sensitive, *P* = 0.033; resistant vs sensitive, *P* = 0.435).

The outcomes of the exploratory analyses examining the trend of CA125 levels are detailed below. In the PP population, we observed a reduction in CA125 after the first cycle of PLD in 39 patients (42.4%). As is shown in Table 5., the ORR was significantly higher in patients with a CA125 decrease after the first cycle than that in the patients with a CA125 increase (66.7% vs.32.1%, *P* = 0.001).

**Table 5:** The predictive impact of a CA125 decrease after the first cycle on the efficacy

A CA125 decrease after the first cycle	YES (*N* = 39, 42.4%)	NO (*N* = 53, 57.6%)	*P*-value^a^
Complete remission, No. (%)	1 (2.6%)	1 (1.9%)	*P* > 0.999
Partial remission, No. (%)	25 (64.1%)	16 (30.2%)	*P* = 0.001
Stable disease, No. (%)	9 (23.1%)	23 (43.4%)	*P* = 0.043
Disease progression, No. (%)	4 (10.3)	13 (24.5%)	*P* = 0.081
ORR^b^, (95%CI)	26 (66.7%)	17 (32.1%)	*P* = 0.001
DCR^c^, (95%CI)	35 (89.7%)	40 (75.5%)	*P* = 0.081

*ORR* Objective response rate, *DCR* Disease control rate; ^a^chi-square test; ^b^Including patients with complete and partial responses, ^c^Including patients with complete and partial responses and stable disease

The numbers of patients in whom efficacy was evaluated by the GCIG criteria are listed in Fig. 1. Totally, efficacy was evaluated by the GCIG criteria in 7 patients, 8 patients and 9 patients after the cycle 2, cycle 4 and cycles 6–8, respectively. Two patients underwent two successive efficacy evaluations by the GCIG criteria. Totally, efficacy was evaluated by the GCIG criteria at least once in 22 patients and by only the RECIST in 70 patients. In terms of the predictive role of baseline CA125, for the 70 patients in whom efficacy was evaluated by the RECIST, the ORR in patients with a low CA125 level at baseline was higher than that in patients without, though the difference was not statistically significant (48.6, 42.9 and 30.0% in patients with baseline CA125 ≤ 200, 200–500 and ≥ 500 U/mL, respectively). For the 22 patients in whom efficacy was evaluated by the GCIG criteria, since the number of patients was not large enough, only a univariate analysis was performed with binary logistic regression, and the predictable role of baseline CA125 was true for these patients (Supplementary Table 2). In terms of the predictive role of a decrease in CA125 after the first cycle, this was true for patients in whom efficacy was evaluated by the RECIST (Supplementary Table 3), and the ORR was higher in patients with a CA125 decrease after the first cycle than that in patients without a CA125 decrease (67.6% vs. 34.7%, respectively, *P* = 0.005). For patients in whom efficacy was evaluated by the GCIG criteria, though the difference was not statistically significant, the ORR was higher in patients with a CA125 decrease after the first cycle (87.5% vs. 42.9%, respectively, *P* = 0.074).

**Fig. 4:**
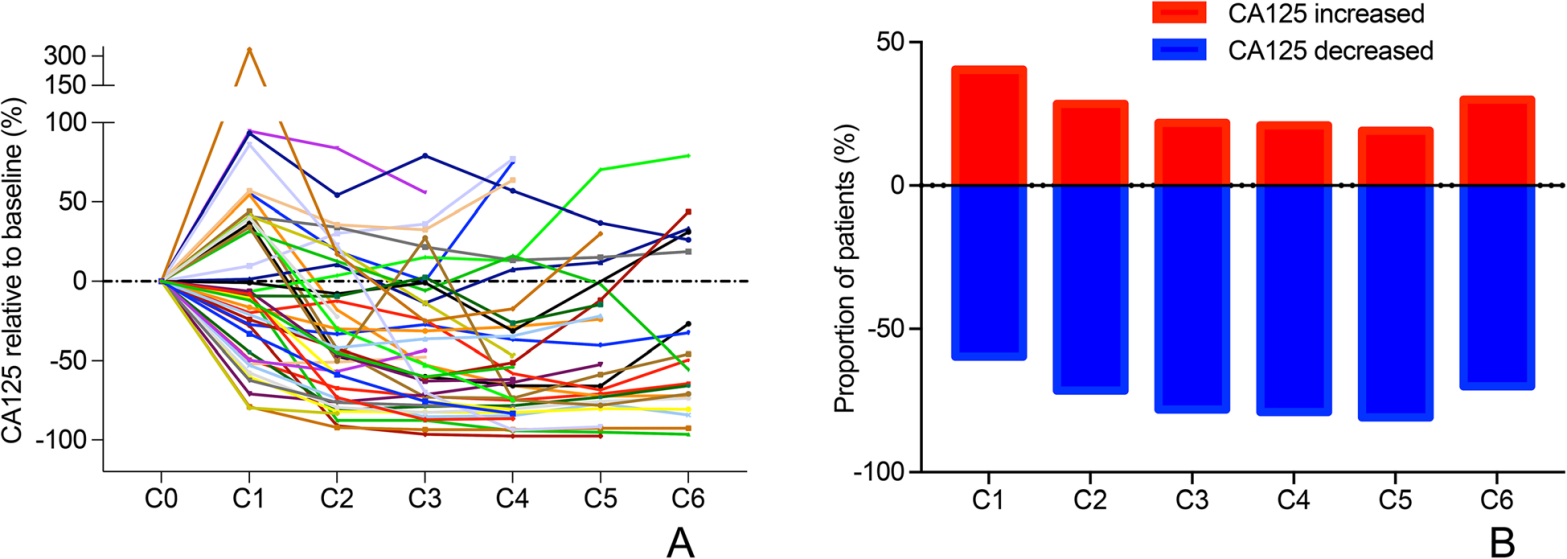
The trend of CA125 relative to baseline in the 43 patients who achieved objective response. **a** The trend of CA125 for each patient. **b** The proportion of patients who had a CA125 increase

The CA125 variations in each patient who achieved an objective response are shown in Fig. 4a. As is shown in Fig. 4b, 40.5, 28.6, 22.0, 21.1, 19.2 and 30.0% of patients who achieved an objective response had increases in CA125 relative to baseline after the cycles 1, 2, 3, 4, 5 and 6, respectively.

### Safety assessment

**Fig. 5:**
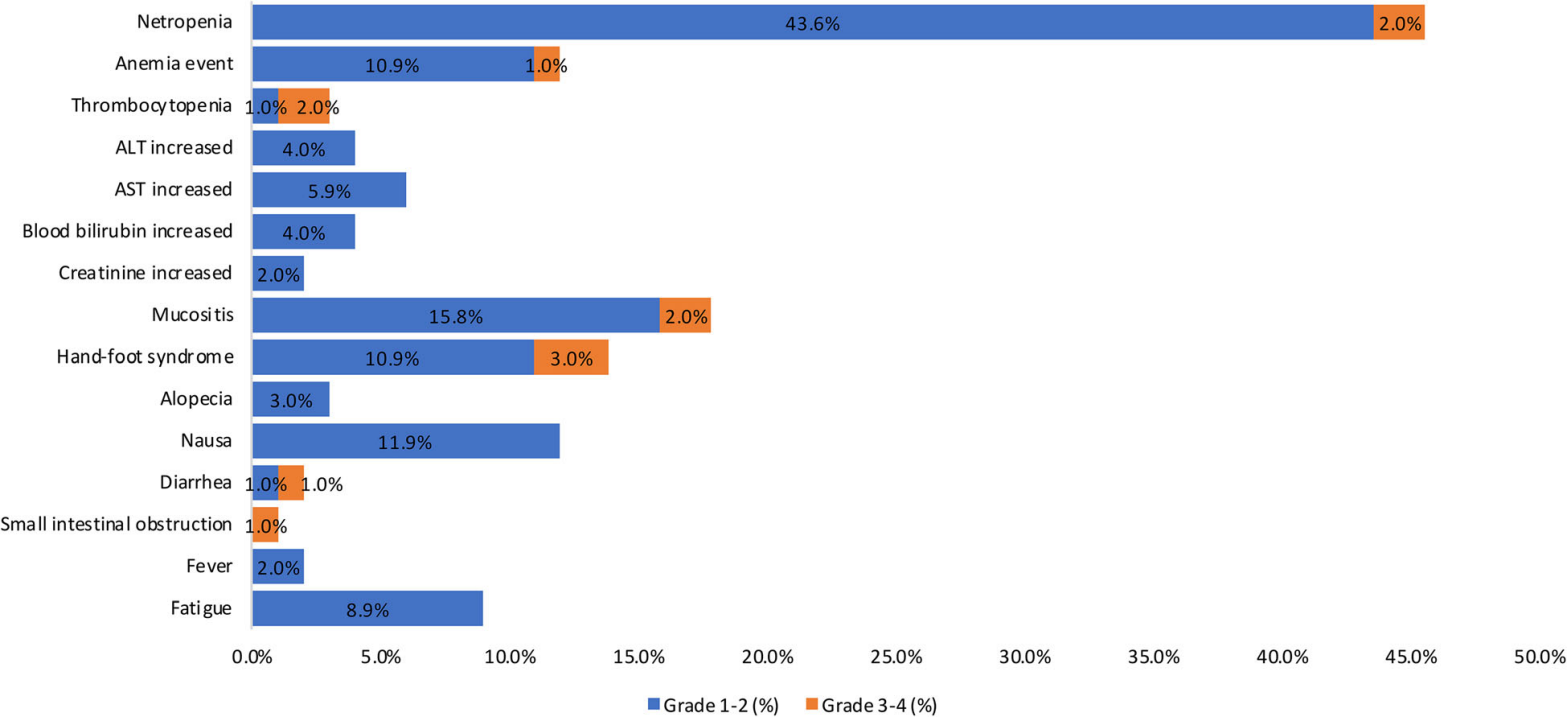
The adverse effects regardless of causality. ALT, alanine aminotransferase; AST, aspartate aminotransferase

As shown in Fig. 5, the most common grade 3 or higher AE regardless of causality was hand-foot syndrome (3 [3.0%] of 101 patients), followed by mucositis (2 [2.0%] of 101 patients), thrombocytopenia (2 [2.0%] of 101 patients), neutropenia (2 [2.0%] of 101 patients), an anemia event (1 [1.0%] of 101 patients) and diarrhea (1 [1.0%] of 101 patients). The most commonly reported all-grade AEs regardless of causality included neutropenia (46 [45.6%] of 101 patients), mucositis (18 [17.8%] of 101 patients), hand-foot syndrome (14 [13.9%] of 101 patients), anemia events (12 [11.9%] of 101 patients) and nausea (12 [11.9%] of 101 patients).

Severe adverse effects regardless of causality were noted in 3 (3.0%) of 101 patients. Of these patients, 1 (1.0% of 101 patients) had a small intestinal obstruction, 1 (1.0% of 101 patients) had a fever due to a peritoneal infection and 1 (1.0% of 101 patients) had a fever due to a viral infection. No patients were reported to have left ventricular systolic dysfunction and no treatment-related deaths were reported. Based on the clinical assessment, the two patients who died before the postbaseline efficacy assessment may have died from disease progression.

### Quality of life assessment

Regarding the QOL assessment, 82 patients (71.9% of 115 patients) completed the QOL questionnaire at baseline, 52 patients (64.2% of 81 patients) before the third cycle of PLD, and 29 patients (64.4% of 45 patients) before the fifth cycle. Regarding the global health status, physical functioning, role functioning, emotional functioning, cognitive functioning and social functioning, higher scores represent better QOL and functioning. For fatigue, nausea/vomiting, pain, appetite, constipation, diarrhea, insomnia, dyspnea and financial problems, higher scores represent worse symptoms. No statistically significant differences existed in any scores between the baseline and the any post-baseline questionnaires (*P* > 0.05, supplementary Fig. 1).

## Discussion

To optimize the use of PLD and avoid unnecessary toxicity effects, predicting responses is of crucial importance [17, 18]. To the best of our knowledge, limited clinical trial studies have investigated the factors for predicting PLD monotherapy activity in patients with recurrent ovarian cancers. Our findings might have important implications for the future management of patients on PLD therapy.

First, we found that a previous response status to first-line platinum-based chemotherapy was a predictive factor of the objective response, which is in line with previous studies [19–21].

Second, and most importantly, we analyzed the predictive role of CA125 levels at baseline and the changes in CA125 after the first cycle of PLD in patients who experienced relative non-platinum-sensitive relapse. We found that a low CA125 level at baseline and a CA125 decrease after the first cycle were predictive factors for a good objective response. Previous studies have shown that an early decline predicts an improved prognosis [22], however, the patients were limited to those patients with platinum-sensitive relapse. For patients with non-platinum- sensitive relapse, there are limited data. Therefore, it remains uncertain whether CA125 levels or variation can be utilized to predict the efficacy in the non-platinum- sensitive setting. The findings of this study showed that CA125 levels may provide important predictive information about the PLD efficacy in patients with non-platinum- sensitive relapse. Efficacy may be more satisfactory for patients with a baseline CA125 level ≤ 200 U/mL or a CA125 decrease after the first cycle than for other patients. And this was true for the patients in whom efficacy was evaluated by the GCIG criteria or the RECIST. Since this was a preliminary analysis and the sample size of patients enrolled was not enough, if the number of patients enrolled increased, the difference may become statistically significant. On the other hand, even for the patients who achieved an objective response, a portion of these patients had a CA125 increase from the baseline after each cycle. The highest proportion was 40.5% which occurred after the first cycle, subsequently, the proportion decreased. This trend is consistent with that observed in a previous study [23]. While bevacizumab might influence CA125 levels by altering the regulation of MUC16 expression [24], the reason why this transient increase in CA125 occurs during the early treatment of PLD needs to be investigated.

The choice of second-line chemotherapy depends on several factors such as the platinum-free interval, persistent side-effects after prior treatments, toxic profiles of future therapies and patient preferences [21]. For platinum-resistant/platinum-refractory relapse, sequential single-agent salvage chemotherapy is superior to multiagent chemotherapy which increases toxicity without clear benefits; however, no priority sequence of these single agents has been recommended [5]. As previously mentioned, PLD is the most common initial therapy in the real world [6]. In this preliminary analysis, though the total sample size was 67 (less than 78), 20 patients had achieved an objective response. Therefore, PLD could be considered a success and the response rate in the platinum-resistant and-refractory group was 29.9%, which is consistent with previous studies that reported response rates of 15% [16], 23.1% [25], 26% [26] or 40.4% [6]. Moreover, the response rate of PLD was similar to that of other single agents: topotecan, 17% [27] -20.5% [28]; gemcitabine, 9.2% [29] -29% [30]; oral etoposide, 26.8% [31]; docetaxel, 22.4% [32] and weekly paclitaxel,13.2% [28] -25% [33].

The median time interval, from the date of enrollment to the date at which a patient was switched to another therapy, was 4 months and 8 months for patients who experienced platinum-refractory relapse and platinum-resistant relapse, respectively. In that many patients in our study switched to other anticancer therapies based on the local investigator’s decision, before disease progression, therefore, this time interval, we analyzed, was shorter than progression-free survival (PFS) which was defined as the time interval from the date of enrollment to the date of disease progression. Even so, the time interval following treatment with PLD, in our study, was comparable to or even longer than the PFS with other single agents in other previous studies: topotecan, 4.7 months [34]; paclitaxel, 3.7 months [34]; gemcitabine, 3.6 months [29]; oral etoposide, 5.7 months [31]; and weekly paclitaxel, 3.49 months [33].

Moreover, 32.3 and 80.7% of patients in our study who experienced platinum-refractory and -resistant relapse, respectively, were able to be retreated with platinum-based chemotherapy; for these patients, the platinum-free interval could be artificially prolonged to 6 months or more using PLD.

In patients with partially sensitive relapse, two options are available: platinum-based doublets or non-platinum therapy (single-agent or combination) [21]. Whether prolonging the platinum-free interval through with a nonplatinum-based chemotherapy agent could improve overall prognosis is controversial. On the one hand, prolonging the platinum-free interval is hypothesized to increase the sensitivity to subsequent retreatment with platinum [3, 8]. Moreover, an in vitro study has demonstrated that extending the platinum-free interval in patients with recurrent ovarian cancer could reverse resistance to platinum [35]. On the other hand, in 2017, the MITO 8 study compared the experimental sequence of a non-platinum single agent chemotherapy followed by a platinum based chemotherapy versus the reversed sequence, and demonstrated that the use of non-platinum-based chemotherapy to artificially prolong the platinum-free interval did not effectively improve prognosis [9].

However, the platinum-based therapy is not always the best option. The incidence of hypersensitivity reactions (allergic reactions) of any grade to carboplatin is approximately 12–19% [36]. The rate of hypersensitivity reactions increases with more frequent exposure to carboplatin, and has been reported to be 27% in cycle 7 or higher [37]. Concerning more severe or life-threatening reactions, unless the patient is under the guidance of a specialist with desensitization experience, the drug should not be used again [38]. Moreover, approximately 50% of patients re-challenged with platinum-based chemotherapy experienced recurrent hypersensitivity reactions despite premedication [39]. The decision to re-challenge should be based on several clinical factors, including the risks for severe recurrent hypersensitivity reactions and the potential clinical benefits of further treatment [36]. Additionally, adverse effects should be carefully taken into account before considering platinum re-challenge; cumulative myelosuppression, characterized by thrombocytopenia, granulocytopenia and anemia, is the main toxicity associated with carboplatin [12]. At the very least, non-platinum-based chemotherapy allows extra time for the patient to recover from toxic effects of their front-line platinum-based therapy [40]. Moreover, a significant proportion of patients with recurrent ovarian cancer are considered “fragile” and therefore not fit to receive further platinum-based chemotherapy treatments due to their poor performance status and/or older age. Therefore, some less toxic options have been suggested for these patients [12]. For patients who experience partially platinum-sensitive relapse, PLD may not be preferred but may be an option for selected patients with an ORR of 47.9% in the ITT population and 56.1% in the PP population. Importantly, we want to emphasize that for patients with partially platinum-sensitive relapse, the choice of non-platinum-based chemotherapy should be prudent, and thoughtful evaluations of the disease status, the performance status of the patient, adverse effects of front-line platinum-based chemotherapy and the planned treatment strategy for the follow-up treatment are essential. Above all, enough consent must be obtained.

The most common treatment-related AEs included myelosuppression, foot-hand syndrome and mucositis [41]. In this study, grade 3–4 myelosuppression included neutropenia (2.0%), anemia (1.0%) and thrombocytopenia (2.0%). Consistent with previous evidence, the myelosuppression observed in our study was generally mild [42, 43]. Therefore, more patients were able to receive other subsequent treatments. Consequently, PLD has a better impact on overall survival than other single agents [26]. In this study, the rates of grade 3–4 ft-hand syndrome and mucositis were 3.0 and 2.0%, respectively. Which is consistent with the previous studies [42, 43]. In some studies, the rates of foot-hand syndrome and mucositis may be higher than those reported in this study [44, 45], and may be due to a higher dose of PLD administered (50 mg/m^2^). Previous studies have shown that the rate of these adverse effects increases with increasing drug doses and decreasing dose intervals [42]. In line with previous reports, the incidence of severe adverse effects was very low, remaining lower than the 4% of the patients treated [43]. We believe that the adverse effects associated with PLD in this study were relatively favorable, even compared with those of oral anticancer therapy agents such as apatinib combined with oral etoposide, which had incidences of 50, 32, 29, and 24% for grade 3 or 4 neutropenia, fatigue, anemia and mucositis, respectively [46], although cross-trial comparisons were difficult.

In addition to the relatively low rate of adverse effects, the QOL did not change significantly during the treatment, and no differences in the QOL-C30 scores were found between baseline and post-chemotherapy. In parallel with the ongoing improvements in cancer treatment options, the effects of treatment on QOL are also important to consider [47]. QOL is especially important in patients with recurrent ovarian cancer, which is generally incurable [8]. Moreover, the 4-week cycle of PLD was well-accepted and more patient-friendly than the 3-week or 1-week cycle of other agents [7]. All of the above findings may support why PLD is the most common initial agent used in the real world for patients with platinum-refractory and platinum-resistant relapse.

## Conclusion

In conclusion, for patients who experience platinum-resistant and refractory relapse, the use of PLD may be a good choice because of the associated satisfactory efficacy, low frequency of adverse effects and high QOL. Moreover, a low CA125 level at baseline and a reduction in CA125 after the first cycle are predictive factors for satisfactory efficacy.

## Supplementary Information

**Additional file 1: Supplementary Table 1**. The efficacy analysis of patients with high-grade serous cancer. ORR, objective response rate; DCR, disease control rate; ^a^ Twelve patients receiving less than 2 cycles of PLD and 4 patients without efficacy assessments after 2 cycles of PLD were excluded. Moreover, 1 patient had a postbaseline efficacy assessment that could not be confirmed and was thus excluded; ^b^ Including patients with complete and partial responses; ^c^ Including patients with complete and partial responses and stable disease.

**Additional file 2:Supplementary Table 2**. Predictive impact of factors on efficacy evaluated by the RECIST or the GCIG criteria. ECOG, Eastern Cooperative Oncology Group; FIGO, International Federation of Gynecology and Obstetrics; RECIST, Response Evaluation Criteria in Solid Tumors version 1.1; GCIG, Gynecologic Cancer InterGroup.

**Additional file 3:Supplementary Table 3**. Predictive impact of a CA125 decrease after the first cycle on efficacy evaluated by the RECIST or the GCIG criteria. ORR, objective response rate; DCR, disease control rate; a chi-square test; b Including patients with complete and partial responses; c Including patients with complete and partial responses and stable disease. RECIST, Response Evaluation Criteria in Solid Tumors version 1.1; GCIG, Gynecologic Cancer InterGroup.

**Additional file 4:** **Supplementary Figure 1** Quality of Life Questionnaire scores.

## Data Availability

The primary data described in this study are available from the authors upon direct request.

## Abbreviations

AEs: Adverse events
CA: Cancer antigen;
CI: Confidence intervals
CR: Complete remission
CT: Computed tomography
CTCAE: Common terminology criteria for adverse events
DCR: Disease control rate
ECOG: Eastern cooperative oncology group
EORTC QLQ-C30: Organization for research and treatment of cancer core quality of life questionnaire
FIGO: International federation of gynecology and obstetrics
GCIG: Gynecologic cancer intergroup
IQR: Interquartile range
ITT: Intention-to-treat.
ORR: Objective response rate
PD: Progressive disease
PLD: Pegylated liposomal doxorubicin
PP: Per-protocol
PR: Partial remission
QOL: Quality of life
RECIST: Response evaluation criteria in solid tumors
SD: Stable disease
