# Supplementary material for: Pegylated liposomal doxorubicin in patients with epithelial ovarian cancer
Source: J Ovarian Res. 2021 Jan 11;14:12. doi: 10.1186/s13048-020-00736-2 (PMC7798203; doi:10.1186/s13048-020-00736-2)
Supplement: Supplementary file 1 — Additional file 1: Supplementary Table 1. The efficacy analysis of patients with high-grade serous cancer. ORR, objective response rate; DCR, disease control rate; a Twelve patients receiving less than 2 cycles of PLD and 4 patients without efficacy assessments after 2 cycles of PLD were excluded. Moreover, 1 patient had a postbaseline efficacy assessment that could not be confirmed and was thus excluded; b Including patients with complete and partial responses; c Including patients with complete and partial responses and stable disease. [file 13048_2020_736_MOESM1_ESM.docx]

Supplementary table 1. The efficacy analysis of patients with high-grade serous cancer. ORR, objective response rate; DCR, disease control rate; ^a^ Twelve patients receiving less than 2 cycles of PLD and 4 patients without efficacy assessments after 2 cycles of PLD were excluded. Moreover, 1 patient had a postbaseline efficacy assessment that could not be confirmed and was thus excluded; ^b^ Including patients with complete and partial responses; ^c^ Including patients with complete and partial responses and stable disease.

|  | **Intention-to-treat population (N=91)** | | | **Per-protocol population (N=74 ^a)^** | | |
| --- | --- | --- | --- | --- | --- | --- |
|  | Platinum-refractory (N=30) | Platinum-resistant (N=25) | Partial platinum-sensitive (N=36) | Platinum-refractory (N=20) | platinum-resistant (N=21) | Partially platinum-sensitive (N=33) |
| Complete remission, No. (%) | 0, (0.0%) | 0, (0.0%) | 2, (5.6%) | 0, (0.0%) | 0, (0.0%) | 2, (6.1%) |
| Partial remission, No. (%) | 3, (10.0%) | 13, (52.0%) | 18, (50.0%) | 3, (15.0%) | 13, (61.9%) | 18, (54.5%) |
| Stable disease, No. (%) | 13, (43.3%) | 3, (12.0%) | 8, (22.2%) | 13, (65.0%) | 3, (14.3%) | 8, (24.2%) |
| Disease progression, No. (%) | 4, (13.3%) | 5, (20.0%) | 5, (13.9%) | 4, (20.0%) | 5, (23.8%) | 5, (15.2%) |
| ORR^b^, (95%CI) | 10.0%  (-1.4%-21.4%) | 52.0%  (31.0%-73.0%) | 55.6%  (38.5%-72.6%) | 15.0%  (-2.1%-32.1%) | 61.9%  (39.3%-84.6%) | 60.6%  （43.0%-78.2%） |
| DCR^c^, (95%CI) | 53.3%  (34.4%-72.3%) | 64.0%  (43.8%-84.2%) | 77.8%  (63.5%-92.0%) | 80.0%  (60.8%-99.2%) | 76.2%  (56.3%-96.1%) | 84.8%  (71.9%-97.8%) |
