# Supplementary material for: Pegylated liposomal doxorubicin in patients with epithelial ovarian cancer
Source: J Ovarian Res. 2021 Jan 11;14:12. doi: 10.1186/s13048-020-00736-2 (PMC7798203; doi:10.1186/s13048-020-00736-2)
Supplement: Supplementary file 2 — Additional file 2:Supplementary Table 2. Predictive impact of factors on efficacy evaluated by the RECIST or the GCIG criteria. ECOG, Eastern Cooperative Oncology Group; FIGO, International Federation of Gynecology and Obstetrics; RECIST, Response Evaluation Criteria in Solid Tumors version 1.1; GCIG, Gynecologic Cancer InterGroup. [file 13048_2020_736_MOESM2_ESM.docx]

| **The impact factors of**  **Objective response** | **RECIST** | | **GCIG** | |
| --- | --- | --- | --- | --- |
|  | **Univariate analysis** | | **Univariate analysis** | |
|  | **P-value** | **OR (95%CI)** | **P-value** | **OR (95%CI)** |
| Age | 0.240 |  | 0.840 |  |
| ECOG | 0.800 |  | 0.769 |  |
| Histology | 0.991 |  | >0.999 |  |
| FIGO stage | 0.336 |  | >0.999 |  |
| Neoadjuvant therapy | 0.729 |  | 0.200 |  |
| Residue tumor | 0.509 |  | 0.993 |  |
| Response status to platinum | 0.119 |  | 0.182 |  |
| Resistant VS. Refractory | 0.075 | 3.657  (0.878-15.242) | 0.326 | 3.600  (0.280-46.359) |
| Partially sensitive VS. Refractory | 0.059 | 3.200  (0.956-10.714) | 0.068 | 18.000  (0.812-399.154) |
| Baseline CA125 | 0.576 |  | 0.030 |  |
| 200-500 VS. ≤200 | 0.671 | 0.792  (0.269-2.327) | 0.028 | 0.050  (0.003-0.719) |
| ≥500 VS. ≤200 | 0.2999 | 0.452  (0.101-2.024) | 0.016 | 0.025  (0.001-0.504) |

Supplementary table 2. Predictive impact of factors on efficacy evaluated by the RECIST or the GCIG criteria. ECOG, Eastern Cooperative Oncology Group; FIGO, International Federation of Gynecology and Obstetrics; RECIST, Response Evaluation Criteria in Solid Tumors version 1.1; GCIG, Gynecologic Cancer InterGroup.
