# Supplementary material for: Pegylated liposomal doxorubicin in patients with epithelial ovarian cancer
Source: J Ovarian Res. 2021 Jan 11;14:12. doi: 10.1186/s13048-020-00736-2 (PMC7798203; doi:10.1186/s13048-020-00736-2)
Supplement: Supplementary file 3 — Additional file 3:Supplementary Table 3. Predictive impact of a CA125 decrease after the first cycle on efficacy evaluated by the RECIST or the GCIG criteria. ORR, objective response rate; DCR, disease control rate; a chi-square test; b Including patients with complete and partial responses; c Including patients with complete and partial responses and stable disease. RECIST, Response Evaluation Criteria in Solid Tumors version 1.1; GCIG, Gynecologic Cancer InterGroup. [file 13048_2020_736_MOESM3_ESM.docx]

Supplementary table 3. Predictive impact of a CA125 decrease after the first cycle on efficacy evaluated by the RECIST or the GCIG criteria. ORR, objective response rate; DCR, disease control rate; a chi-square test; b Including patients with complete and partial responses; c Including patients with complete and partial responses and stable disease. RECIST, Response Evaluation Criteria in Solid Tumors version 1.1; GCIG, Gynecologic Cancer InterGroup.

| CA125 decrease after the first cycle  (RECIST, N=70) | NO  (N=39, 55.7%) | YES  (N=31, 44.3%) | P-value^a^ |
| --- | --- | --- | --- |
| Complete remission, No. (%) | 1 (2.6%) | 1 (3.2%) | P>0.999 |
| Partial remission, No. (%) | 10 (25.6%) | 18 (58.1%) | P=0.006 |
| Stable disease, No. (%) | 15 (38.5%) | 8 (25.8%) | P=0.263 |
| Disease progression, No. (%) | 13 (33.3%) | 4 (12.9%) | P=0.048 |
| ORR^b^, (95%CI) | 17 (34.7%) | 23 (67.6%) | P=0.005 |
| DCR^c^, (95%CI) | 36 (73.5%) | 30 (88.2%) | P=0.048 |
| CA125 decrease after the first cycle  (GCIG, N=22) | NO  (N=14, 63.6%) | YES  (N=8, 36.4%) | P-value^a^ |
| Complete remission, No. (%) | 0 (0.0%) | 0 (0.0%) | P>0.999 |
| Partial remission, No. (%) | 6 (42.9%) | 7 (87.5%) | P=0.074 |
| Stable disease, No. (%) | 8 (57.1%) | 1 (12.5%) | P=0.074 |
| Disease progression, No. (%) | 0 (0.0%) | 0 (0.0%) | P>0.999 |
| ORR^b^, (95%CI) | 6 (42.9%) | 7 (87.5%) | P=0.074 |
| DCR^c^, (95%CI) | 14 (100.0%) | 8 (100.0%) | P>0.999 |
