# Supplementary figures and images for: Pegylated liposomal doxorubicin in patients with epithelial ovarian cancer
Source: J Ovarian Res. 2021 Jan 11;14:12. doi: 10.1186/s13048-020-00736-2 (PMC7798203; doi:10.1186/s13048-020-00736-2)

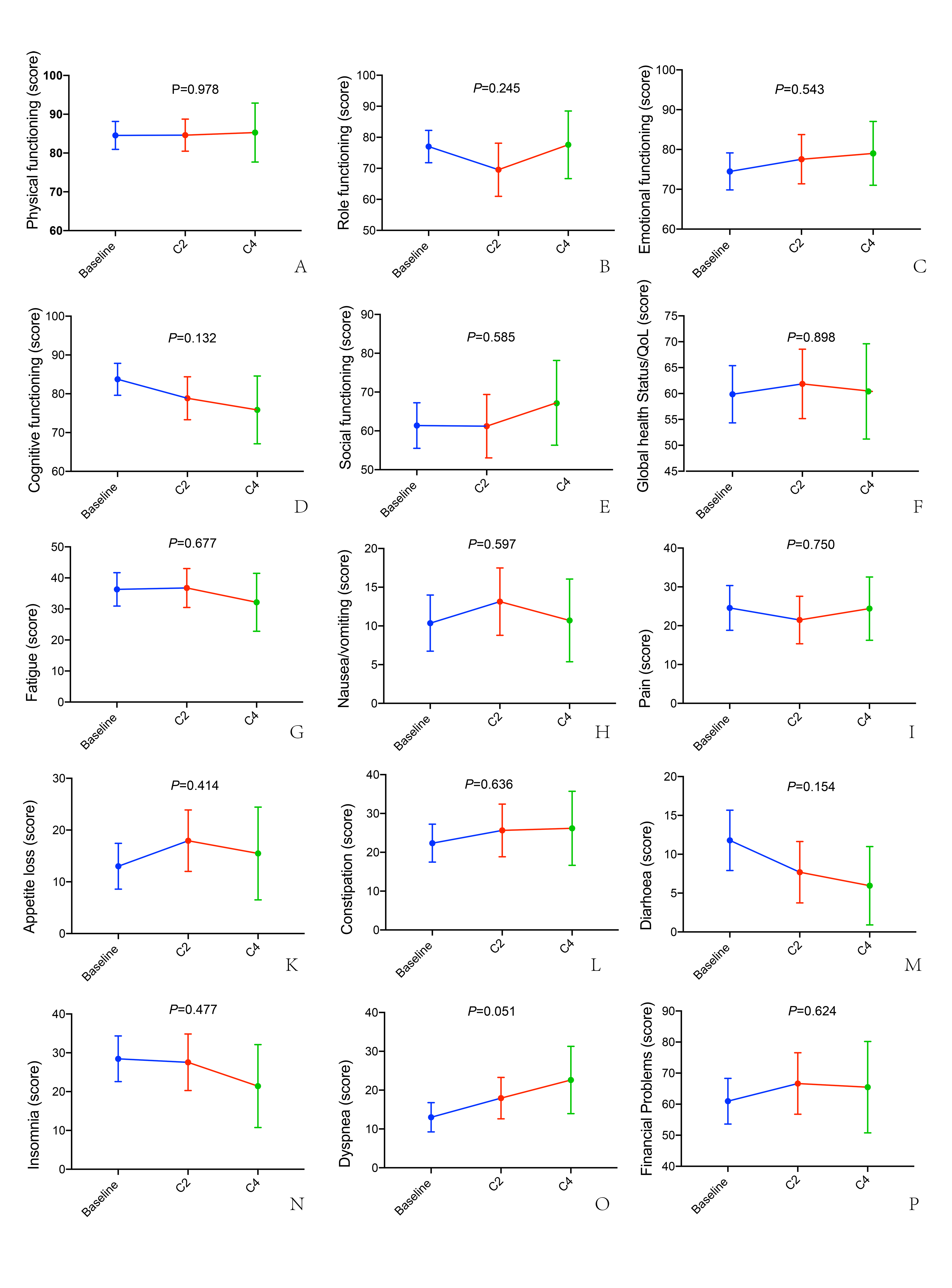

Supplement: Supplementary file 4 — Additional file 4: Supplementary Figure 1 Quality of Life Questionnaire scores. [file 13048_2020_736_MOESM4_ESM.tif]
